# *Plasmodium falciparum* transcription factor AP2-06B is mutated at high frequency in Southeast Asia but does not associate with drug resistance

**DOI:** 10.3389/fcimb.2024.1521152

**Published:** 2025-01-06

**Authors:** Qiyang Shi, Changhong Wang, Wenluan Yang, Xiaoqin Ma, Jianxia Tang, Jiayao Zhang, Guoding Zhu, Yinlong Wang, Yaobao Liu, Xiaoqin He

**Affiliations:** ^1^ National Health Commission Key Laboratory of Parasitic Disease Control and Prevention, Jiangsu Provincial Key Laboratory on Parasite and Vector Control Technology, Jiangsu Institute of Parasitic Diseases, Wuxi, China; ^2^ Laboratory of Molecular Parasitology, State Key Laboratory of Cardiology and Research Center for Translational Medicine, Shanghai East Hospital, Clinical Center for Brain and Spinal Cord Research, School of Medicine, Tongji University, Shanghai, China

**Keywords:** drug-resistant, AP2-06B, *pfk13*, *pfcrt*, AP2

## Abstract

**Introduction:**

A continuing challenge for malaria control is the ability of *Plasmodium falciparum* to develop resistance to antimalarial drugs. Members within the *Plasmodium* transcription factor family AP2 regulate the growth and development of the parasite, and are also thought to be involved in unclear aspects of drug resistance. Here we screened for single nucleotide polymorphisms (SNPs) within the AP2 family and identified 6 non-synonymous mutations within AP2-06B (PF3D7_0613800), with allele frequencies greater than 0.05. One mutation, K3124R, was located in a PfAP2-06B AP2 domain.

**Methods:**

To investigate transcriptional regulation by PfAP2-06B, ChIP-seq assays were performed on 3D7/PfAP2-06B-GFP schizonts using antibodies against GFP. The DNA sequences of the artemisinin-resistant CWX and the quinoline-resistant strains PfDd2 and Pf7G8 were analyzed for the genetic diversity of AP2-06B, compared with the Pf3D7 strain as a reference sequence. To determine whether AP2-06B can alter the expression of *pfk13* and *pfcrt*, as well as cause artemisinin and quinoline resistance in *Plasmodium*, we generated both a K3124R mutation and conditional knockdown of AP2-06B in Pf3D7 using CRISPR/Cas9-mediated genome editing.

**Results:**

ChIP-Seq analysis showed that AP2-06B can bind to the loci of the *Plasmodium* genes *pfk13* and *pfcrt*. The AP2-06B K3124R mutation was also found in the artemisinin-resistant parasite strain CWX and the chloroquine-resistant strains Dd2 and 7G8. Contrary to expectation, Pf3D7 *Plasmodium* lines modified by either K3124R mutation of AP2-06B or conditional knockdown of AP2-06B did not have altered sensitivity to artemisinin or quinolines by modulating *pfk13* or *pfcrt* expression.

**Discussion:**

AP2-06B was predicted to be associated with artemisinin and quinoline resistance, but no change in resistance was observed after mutation or conditional knockdown. Given the multigenic nature of resistance, it might be difficult to recreate a resistance phenotype. In conclusion, whether AP2-06B regulates the development of artemisinin or quinoline resistance remains to be studied.

## 1 Introduction

The parasitic disease malaria remains a global public health problem, and in 2022 there were an estimated 249 million cases in 85 endemic countries and regions, according to the World Health Organization (WHO) (World Health Organization, 2023). Falciparum malaria, caused by *Plasmodium falciparum*, is the main cause of malaria deaths. *P. falciparum* has developed degrees of resistance to all widely used antimalarial drugs; thus presenting a challenge for malaria control. To provide the necessary cure rates and to delay the onset of resistance, artemisinin-based combination therapies (ACTs) are recommended by the WHO (Wicht et al., 2020). ACTs have been successful in controlling malaria and have saved countless lives, with the global burden showing a 37% reduction from 2000 to 2015 (Wicht et al., 2020). These gains, however, are threatened by the rise of *P. falciparum* resistance to artemisinin and ACT partner drugs (Blasco et al., 2017; Phillips et al., 2017).

Quinolines are traditional antimalarial drugs, and include the 4-aminoquinoline derivatives chloroquine, piperaquine, amodiaquine, and 8-aminoquinoline derivatives such as primaquine (World Health Organization, 2020). Quinolines are widely used as partner drugs in the first-line therapy ACTs for falciparum malaria; such as dihydroartemisinin-piperaquine and artesunate-amodiaquine, which are employed as first-line drugs in Africa and Southeast Asia (Blasco et al., 2017). However, resistance to artemisinins and partner drugs is now causing the failure of *P. falciparum* ACTs in southeast Asia (Amaratunga et al., 2016; Thanh et al., 2017; Corrections, 2018; Wicht et al., 2020; Ye et al., 2022). Of great concern is the rapid spread of resistance to the ACT dihydroartemisinin-piperaquine, which has been the first-line treatment and the preferred ACT in most of Southeast Asia (Duru et al., 2015; Leang et al., 2015; Spring et al., 2015; Phuc et al., 2017).

Resistance to artemisinin and loss of potency in turn causes a greater reliance on partner drugs to work quickly and effectively. The parasite *k13* gene (*kelch13*) is a genetic determinant of artemisinin resistance, and was first identified in a laboratory-based *in vitro* evolution study (Ariey et al., 2014). A GWAS by several groups also implicated *k13* as a candidate marker of clinical artemisinin resistance (Cheeseman et al., 2012; Miotto et al., 2015). K13 mutations have been thought to mediate artemisinin resistance in intra-erythrocytic ring stage parasites primarily via the reduced activation of artemisinin drugs and/or an enhanced parasite capacity to remove damaged proteins. Lowered K13 levels resulting from mutations have been postulated to lead to reduced hemoglobin endocytosis and catabolism in young rings, resulting in lowered levels of free Fe(II)PPIX available to activate artemisinin (Birnbaum et al., 2020). However, no change in the expression of *k13* transcript abundance was detected in K13 mutant clinical isolates (Mok et al., 2015). In addition, reduced expression of *k13* in rings was reported to result in hypersensitivity to artemisinin, as measured using 72-h dose-response assays (not RSAs) in *piggy*Bac transposon-generated mutant parasite lines (Gibbons et al., 2018; Zhang et al., 2018), contrary to the expected increased tolerance. Further work is required to evaluate whether K13 protein levels or functionalities are affected across all clinical isolates bearing mutations that associate with clinical resistance to artemisinin.

Reduced efficacy in ACT therapies is largely due to the resistance of malaria parasites to the partner drugs (Haldar et al., 2018). For example, in Cambodia, Thailand, and Vietnam piperaquine resistance has led to local malaria treatment failure rates of more than 50% (van der Pluijm et al., 2019); and therefore, resistance to quinolines is of concern (Spring et al., 2015). It is generally believed that the *P. falciparum* chloroquine resistance transporter (*pfcrt*) is related to the resistance mechanism of chloroquine and other quinolines. *Pfcrt* is a 3.1 kb gene with 13 exons and encodes a multi-transmembrane protein belonging to a drug metabolite transporter family (Acharya et al., 2024). The PfCRT transporter protein localizes to the digestive vacuole (DV) membrane, consistent with its role in mediating chloroquine efflux out of the DV away from its heme target (Wicht et al., 2020). *P. falciparum* parasites resistant to chloroquine, amodiaquine, or piperaquine all harbor mutations in *pfcrt*. Mutations of *pfcrt* encompassing codons 72–76 are the key marker for *P. falciparum* chloroquine resistance; especially a lysine to threonine mutation at position 76, which affects the drug transport ability of PfCRT and leads to decreased drug concentration in the parasite DV. Knock-down of *pfcrt* reverts drug sensitivity of resistant parasites to chloroquine and desethyl amodiaquine (the active metabolite of amodiaquine) (Wendler et al., 2014). Therefore, mutations and the expression changes of *pfcrt* can lead to changes in susceptibility to chloroquine and other quinolines. Another factor that plays a role in resistance to heme-targeting antimalarials is the P-glycoprotein homolog PfMDR1, encoded by the *P. falciparum* multidrug resistance 1 transporter gene *pfmdr1* (Wicht et al., 2020). Like PfCRT, PfMDR1 lies within the DV membrane, but transport is predicted to be inwardly directed toward the DV. Reducing *pfmdr1* copy number increases parasite susceptibility to mefloquine, halofantrine, lumefantrine, quinine, and artemisinin derivatives (Sidhu et al., 2006).

In eukaryotes, gene expression is often regulated by multiple transcription factors. AP2 proteins are the largest identified family of apicomplexan transcription factors; and 27 members of the AP2 family have been identified in the *Plasmodium falciparum* genome (Jeninga et al., 2019). Growing evidence suggests that the AP2 family of DNA binding proteins regulate the various stages of growth and development of the *Plasmodium* parasite. For example, AP2-G controls sexual commitment (Josling et al., 2020), whilst AP2-I regulates merozoite invasion of erythrocytes in *P. falciparum* (Santos et al., 2017). In other species, such as fungi, transcriptional regulators have been found to cause multiple drug resistance by upregulating the expression of transporters such as ABC or MDR1 (Paul and Moye-Rowley, 2014). In a genome-wide association study it was reported that mutations in *P. falciparum* AP2 were found to be associated with resistance to the antimalarial drug quinine (Wendler et al., 2014); and mutations in AP2 were found in studies of the mechanism of malaria resistance to three antimalarial compounds (Cowell et al., 2018). A study by Cowell et al. identified several resistance mediators that are likely to play roles in parasite transcriptional response to drugs (Cowell et al., 2018). The most commonly mutated genes identified in the study were genes encoding the apicomplexan AP2 family of transcription factors; and the most prominent was encoded by PF3D7_0613800 (AP2-06B). AP2-06B had codon deletions or changes in response to three independent compounds (MMV665882, MMV665939, and MMV011438), out of 73 compounds selected for chemogenetic characterization of antimalarial drug targets and resistance genes (Cowell et al., 2018). In addition, variants in the gene of AP2-06B were associated with resistance to quinine in genome-wide association studies (Wendler et al., 2014). In conclusion, AP2-06B is a candidate transcription factor involved in the regulation of drug resistance in *Plasmodium*.

AP2-06B is a 4109 aa long protein in *P. falciparum* with three putative DNA-binding AP2 domains within the carboxy terminal region of the protein. In our research, K3124R was identified as a non-synonymous mutation of high allele frequency located in one of the AP2 domains of AP2-06B. The mutation frequency of K3124R was observed to be higher in Southeast Asia, where origins of resistance are significantly more frequent than in other regions. Therefore, we predicted that AP2-06B is involved in the development of drug resistance of *P. falciparum* in Southeast Asia. K3124R mutations were found in the AP2-06B domain of artemisinin-resistant field strains CWX which we identified before (Lu et al., 2017); as well as the chloroquine-resistant strains PfDd2 and Pf7G8. To verify whether AP2-06B regulates the expression of genes related to drug resistance in *Plasmodium*, and thus produces drug resistance, we generated both a K3124R mutation and conditional knockdown of AP2-06B in Pf3D7. However, our results suggested that neither parasite lines modified with AP2-06B had altered *Plasmodium* sensitivity to DHA or quinolone.

## 2 Materials and methods

### 2.1 Screening of PfAP2-06B gene SNPs of *P. falciparum*

VCF (Variant Call Format) files were downloaded from the MalariaGEN database (https://www.malariagen.net/resource/26), which includes 7113 *Plasmodium falciparum* samples from 29 countries. To control the quality of samples, the substandard samples were excluded by the following criteria: (1) duplicate samples, (2) samples with low sequencing coverage, (3) samples with unclear geographic origin information, (4) laboratory strains, (5) samples with genotype missing rates >30%, and (6) samples with Fws values (within-host diversity) <0.95. PfAP2-06B gene variants having single nucleotide polymorphisms (SNPs) were screened using bcftools after quality control of the samples, and high-quality SNPs were selected according to the following criteria: (1) exclusion of centromeric, core high-variation, subtelomere repeat, subtelomere high-variation regions, and VQSLOD <0; (2) QUAL>30; and (3) SNP genotype missing rate <20% and (4) biallelic SNPs in coding regions.

### 2.2 Population genetic analysis of the *pfap2-06b* gene

Population-level genetic diversity was characterized by expected heterozygosity (*H_E_*). *H_E_* was calculated using the formula, *H_E_* = [n/(n − 1)][1 − Σp^2^], where n is the number of genotyped samples and p is the frequency of each allele at a locus. Neutral evolutionary history of the *pfap2-06b* gene was determined using Tajima’s D test. The pair-wise genetic differentiation between different regions was estimated using the fixation index (*F_ST_*). All calculations were undertaken using DnaSP (v6.12.03) software.

### 2.3 Parasite cultures

The *Plasmodium falciparum* 3D7 strain was cultured in O type fresh human erythrocytes as described; in complete RPMI 1640 medium (Gibco) with 0.5% Albumax I (Invitrogen) and a gas phase maintained under 5% CO2, 5% O2 and 90% N2 at 37°C (Fan et al., 2020). Parasites were regularly synchronized with repeated 5% sorbitol treatments at the ring stage. For growth curve assays, parasites were tightly synchronized to a 5 h window by purification of schizont stages using Percoll-sorbitol gradients (70% Percoll and 40% Percoll) followed by 5% sorbitol treatment 5 h later (Shang et al., 2022).

### 2.4 Plasmid construction

The pL6cs-ap2-06b-ty1-gfp, pL6cs*-pfap2-06b^K3124R^*, and pL6cs-*ap2-06b-2xfkbp-gfp-2xfkbp* plasmids were constructed as described (Ghorbal et al., 2014; Zhao et al., 2020). First, the guide RNA was annealed by complementary oligonucleotides and cloned into the pL6cs construct between XhoI and AvrII restriction enzyme sites. Then the 3’UTR region of *pfap2-06b* with ty1-GFP, the region of *pfap2-06b* with mutation, or the 3’UTR region of *pfap2-06b* with 2×fkbp-gfp-2×fkbp were cloned into AflII and AscI restriction sites. The constructed plasmids were verified by sequencing and transformed into *Escherichia coli* XL10 for amplification and purification. All primers used for construction are listed in **Supplementary Table S1**.

### 2.5 Generation of transgenic parasite lines

Transfections were performed in uninfected red blood cells using 100 μg of purified pL6cs-*pfap2-06b-ty1-gfp* or pL6cs*-pfap2-06b^K3124R^* plasmid together with 100 μg of pUF1-Cas9-BSD, for the pL6cs-*pfap2-06b-2xfkbp-gfp-2xfkbp* plasmid, together with 100 μg of pUF1-Cas9-DSM1 and pLyn-FRB-mCherry plasmids followed by the addition of purified schizont stage parasites (Zhao et al., 2020). Subsequently, parasites were cultured in the presence of 2.5 nM WR99210 and 2 μg/ml BSD or 1.5 μM DMS1 (Invitrogen) until live parasites were identified in Giemsa’s solution-stained thin blood smears roughly 3 weeks later. The sequences at the designed integration sites were examined by PCR using genomic DNA template followed by DNA sequencing to confirm the presence of genetic editing. Clones of the transgenic parasite lines PfAP2-06B-GFP, PfAP2-06B^K3124R^, and PfAP2-06B-KS were derived by limiting dilution cloning (Fan et al., 2020). Primers used for verification are provided in **Supplementary Table S2**.

### 2.6 Growth curve assays

Parasites were tightly synchronized to a 5 h window. Ring-stage parasites were plated at 0.1% parasitemia in a 6-well plate with 2% hematocrit fresh erythrocytes. For PfAP2-06B^K3124R^ parasites, the wild-type strain 3D7 was used as a control group. PfAP2-06B-KS parasites were cultured in the presence or absence of rapalog, a ligand that causes FRB and FKBP to form dimers, and thereby remove the target protein from its site of action to achieve knockdown effect (Birnbaum et al., 2017). Parasitemias were monitored periodically by counting trophozoite-stage parasites within Giemsa-stained thin blood smears for three intraerythrocytic growth cycles (Shang et al., 2021).

### 2.7 Ring survival assay

RSAs were performed as described (Witkowski et al., 2013; Zhang et al., 2017; Siddiqui et al., 2018). Briefly, highly synchronous parasite cultures at the young ring stage (0 to 3 h) were exposed for 6 h to 700 nM DHA or 0.1% dimethyl sulfoxide (DMSO) as the control. The drug was then washed out with RPMI 1640, and parasites were further cultivated further for 66 h under standard *in vitro* culture conditions. At 72 h after the assay initiation, survival rates were calculated in Giemsa-stained thin smears by counting the viable parasites surviving in DHA-treated versus DMSO-treated cultures. Parasite isolates demonstrating >1% survival were considered to display reduced susceptibility, or partial resistance, to artemisinin (Amaratunga et al., 2014).

### 2.8 Immunofluorescence assays

Immunofluorescence assays were performed as described to characterize PfAP2-06B localization (Shang et al., 2021). Parasites were harvested at the schizont stage and fixed by 4% paraformaldehyde (Electron Microscopy Sciences) at room temperature for 10 min, then washed with PBS. Prepared samples were incubated with primary antibody against GFP (Proteintech, 66002-1-Ig) at 1:500 to 1:1,000, followed by a secondary antibody AlexaFluor 488 goat anti-mouse IgG (ThermoFisher Scientific, A11029) at 1:500.The samples were then incubated with rabbit anti-mCherry (Proteintech, 26765-1-AP) at 1:300, followed by secondary antibody AlexaFluor 568 goat anti-rabbit IgG (ThermoFisher Scientific, A11036) at 1:500. For nuclear localization, the samples were washed with PBS and incubated with DAPI for 30 min. Preparations were visualized with a Nikon A1R microscope at 60-100× magnification, and images were acquired with NIS Elements software and processed using Adobe Photoshop.

### 2.9 The susceptibility test of parasites to quinolines

To determine the sensitivity of parasites to quinolines, IC50 assays of 3-day inhibition tests were performed using chloroquine or amodiaquine. Briefly, 200 μL of a tightly synchronized parasite culture at 1% parasitemia and 2% hematocrit was added into each well of a 96-well plate, followed by chloroquine or amodiaquine drug pressure at a series of concentrations (500 nmol/L, 250 nmol/L, 125 nmol/L, 62.5 nmol/L, 31.25 nmol/L, 15.63 nmol/L, 7.81 nmol/L, and 3.91 nmol/L). After 72 hours of incubation, the parasiticidal effects of the drugs were estimated by the addition of 100 μL of SYBR Green I lysis buffer to each well. After thorough mixing, the plates were incubated for 1 h and then SYBR Green fluorescence values were detected using a microplate reader (490 nm excitation and 530 nm emission wavelengths). IC50 values were calculated by GraphPad Prism. The formulation of SYBR Green I lysis buffer solution is listed in **Supplementary Table S3**.

### 2.10 Quantitative reverse PCR

Highly synchronous ring (10–16 hpi), trophozoite (24–30 hpi), and schizont (40–46 hpi) stage parasites were collected in TRIzol. Total RNA purification was achieved using a Direct-zol RNA Kit (Zymo Research). To assay the levels of specific transcripts, 500–800 ng of total RNAs were reverse transcribed (Takara), and quantitative PCR assays were performed in triplicate using the primers shown in **Supplementary Table S4** and *seryl-tRNA synthetase* (PF3D7 0717700) as the endogenous control. PCR thermal cycling was initiated with 30 sec denaturation at 95°C, followed by 40 cycles of 5 sec at 95°C, 20 sec at 54°C, 7 sec at 56°C, 7 sec at 59°C, and 27 sec at 62°C. Gene expression changes were quantified using the ^△△^Ct method.

### 2.11 Chromatin immunoprecipitation sequencing

ChIP-Seq assays were carried out in two biological replicates as described, with minor modifications (Santos et al., 2017; Josling et al., 2020). Synchronized parasites were harvested at the schizont stage and cross-linked immediately with 1% paraformaldehyde (Sigma) by rotating for 10 min at 37°C, then quenched with 0.125 M glycine for 5 min on ice. The parasites were resuspended in 50 ml of PBS and lysed with 0.15% saponin for 5 min on ice. The released nuclei were washed several times with PBS, then sonicated using an M220 sonicator (Covaris) at 5% duty factor, 200 cycles per burst, and 75W of peak incident power to generate 100-500 bp fragments. The samples were diluted tenfold with dilution buffer and precleared with Protein A/G magnetic beads (Thermo) for 2 hours at 4°C. The precleared chromatin supernatants, a small part of which were aliquoted as input controls, were incubated overnight at 4°C with 0.5 μg of antibodies against GFP (Abcam, ab290) and 20 μL of Protein A/G magnetic beads. The immunoprecipitates were washed with low salt wash buffer, high salt wash buffer, LiCl wash buffer, and TE buffer, then eluted with Elution Buffer. To reverse the cross-link, the eluted samples were incubated overnight at 45°C and treated with RNase A at 37°C for 30 min and Proteinase K at 45°C for 2 hours. Finally, DNA was extracted using a MinElute PCR purification kit (Qiagen, 28006). For library preparation, 1.5 ng of ChIP DNAs were end-repaired (Epicentre No. ER81050), processed with a protruding 3’ A base (NEB No. M0212L), and ligated with adapters (NEB No. M2200L). Agencourt AMPure XP beads (Beckman Coulter) were then used for size selection and purification. Libraries were amplified using a KAPA HiFi PCR Kit (KAPA Biosystems, KB2500) with the following program: 1 min at 98°C; 12 cycles of 10 s at 98°C and 1 min at 65°C; and finally, extension for 5 min at 65°C. Library sequencing was conducted on an Illumina HiSeq Xten platform and generated 150 bp pair-end reads.

### 2.12 ChIP-seq analysis

To remove residual adapters and low-quality bases, read trimming was conducted with Trimmomatic (Bolger et al., 2014) using a 4 bp window and average window quality above 15. Clipped reads with a minimum length of 50 bp and average read quality above 20 were mapped to the *P. falciparum* 3D7 genome build 47 using Bowtie2 (Langmead and Salzberg, 2012; Bolger et al., 2014) and default parameters. Peaks were identified using the call-summits option of the MACS2 callpeak function (Zhang et al., 2008) and a q-value cutoff of 0.05. Log2-transformed ChIP/input fold enrichment signals were calculated with the MACS2 bdgcmp function and visualized with Gviz (Hahne and Ivanek, 2016). Genomic Ranges (Lawrence et al., 2013) assigned peaks to nearby target genes if they overlapped 5′ UTRs (<3 kb upstream of the translation start sites), gene body, and 3′ UTRs (<0.5 kb downstream of the translation stop sites). Functions enriched in the target genes were analyzed using malaria parasite metabolic pathways (MPMP) (Ginsburg, 2006), functional gene families, and cluster Profiler (Yu et al., 2012) [Benjamini-Hochberg (BH) adjusted p-value of <0.01]. Peaks were extended +/− 250 bp around summits. Those detected in both biological replicates were reserved for discovery of PfAP2-06B DNA binding motifs between 6 bp and 10 bp using DREME (Bailey, 2011) as compared with random genomic regions of 500 bp.

### 2.13 Statistical analysis

All data are reported as mean ± the standard deviation (SD) of three duplications. Statistical differences between multiple groups were analyzed using Student’s T test. Statistical analysis was completed using GraphPad Prism software (version 8.0.1, San Diego, CA, USA). Result with P < 0.05 was defined as statistically significant.

## 3 Results

### 3.1 K3124R is a high frequency mutation located in an AP2-06B AP2 domain in Southeast Asia

**Figure 1:**
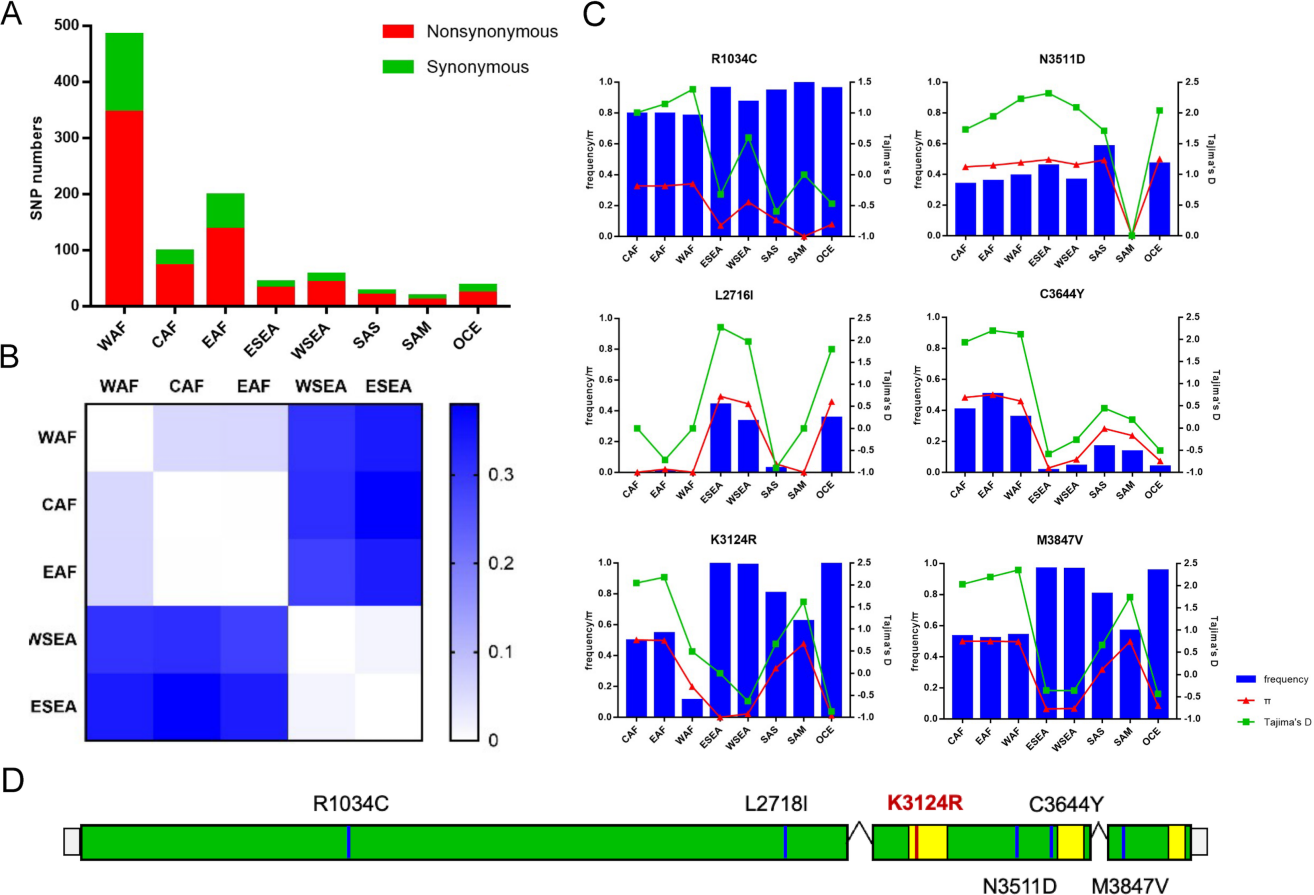
Population genetics of the *pfap2-06b* gene of *P. falciparum*. **(A)** The number of non-synonymous mutations in different global regions. **(B)** Genetic differentiation between Africa and Southeast Asia. **(C)** Six non-synonymous mutations with allele frequency greater than 0.05. **(D)** The location distribution of six non-synonymous mutations on the *pfap2-06b* gene, where the yellow region is a predicted DNA-binding domain of AP2-06B. WAF, West Africa; CAF, Central Africa; EAF, East Africa; WSEA, Southeast Asia (West); ESEA, Southeast Asia (East).

To explore the genetic diversity and population genetics of *P. falciparum* AP2-06B in different geographical strains, the AP2 gene sequences of *P. falciparum* in the MalariaGEN database were analyzed. After sample quality control, 3683 samples were included in this study; specifically, 1,156 West African, 151 Central African, 382 East African, 808 western Southeast Asian, 955 eastern Southeast Asian, 36 South Asian, 37 South American, and 158 from Oceania. In the *pfap2-06b* gene (PF3D7_0613800), 733 SNPs were identified, including 516 non-synonymous mutations and 217 synonymous mutations. The number of non-synonymous mutations observed in African samples was higher than that in other regions, and the highest number of non-synonymous mutations was in West Africa (n=345) (**Figure 1A**). A higher genetic differentiation for *pfap2-06b* was found between Africa and Southeast Asia (*F_ST_* > 0.25) than the respective differentiations within these two regions (**Figure 1B**). There were 22 SNPs with *F_ST_* >0.25 in different regions, among which S256P, L2716I, F2768C, K3124R, N3511D, C3644Y, and M3847F had higher genetic differentiation between Africa and Southeast Asia. The allele frequencies of 6 non-synonymous mutations (R1034C, L2716I, K3124R, N3511D, C3644Y, and M3847V) in PfAP2-06B were greater than 0.05 (**Figure 1C**). Of these, only K3124R was in one of the three putative AP2 DNA-binding domains within PfAP2-06B, which spans approximately aa 3087 to 3136 (**Figure 1D**). K3124R is the mutation located in an AP2-06B domain, and has a significantly higher mutation frequency in Southeast Asia than in other regions.

### 3.2 PfAP2-06B has a significant binding peak in the loci of *pfcrt* and *k13*

**Figure 2:**
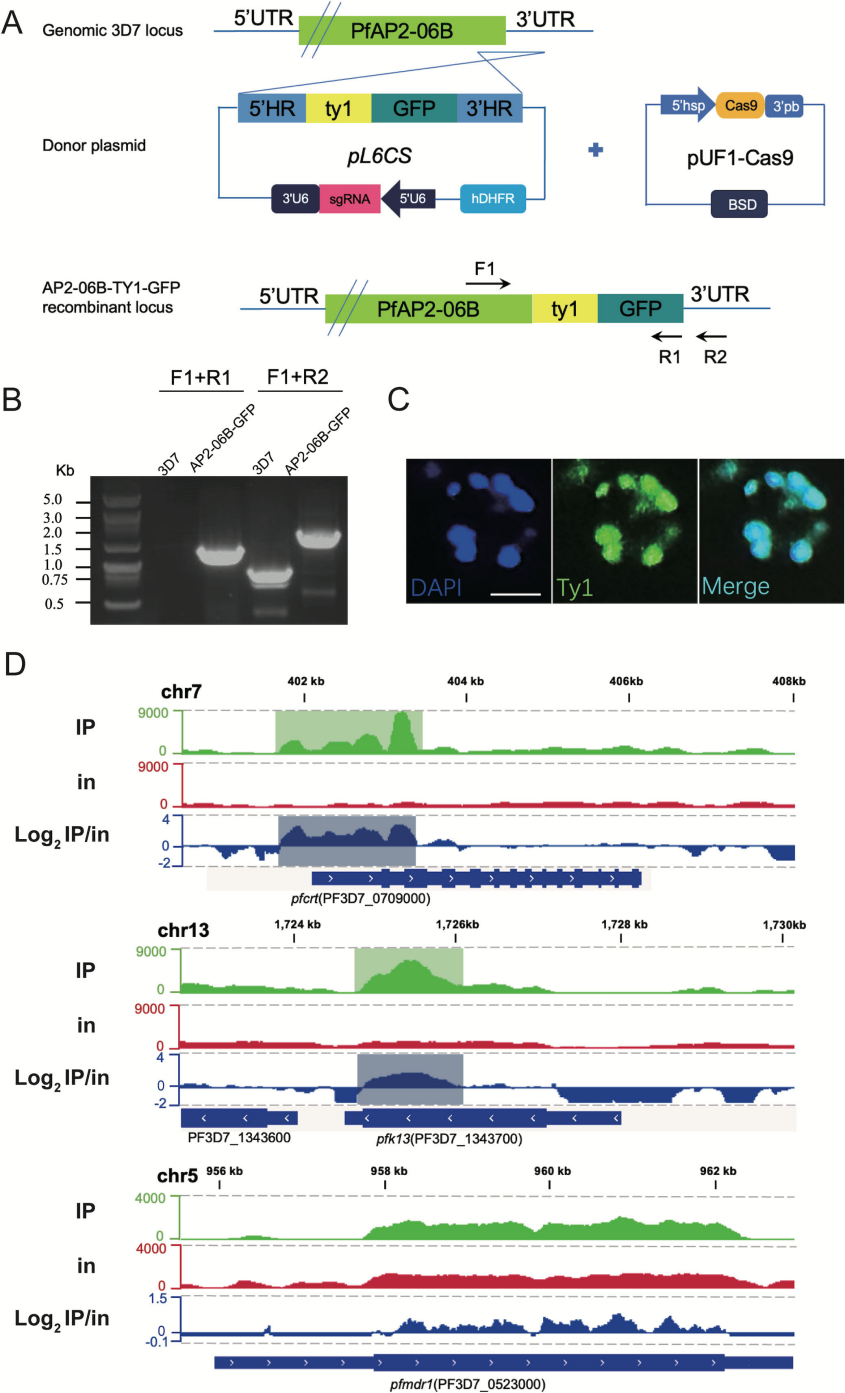
ChIP analysis of the distribution of PfAP2- R at the *pfcrt* gene locus. **(A)** Generation of a *pfap2-06b* transgenic parasite line tagged with TY1-GFP at the C-terminus using the CRISPR/Cas9 gene editing system. **(B)** The TY1-GFP tagged PfAP2-06B transgenic line was verified by diagnostic PCR using F1, R1, and R2 primers. **(C)** Representative fluorescence microscopy images of PfAP2-06B-GFP late stage schizonts. Immunofluorescence assays using anti-TY1 revealed perinuclear distribution of PfAP2-06B in the 3D7/PfAP2-06B- GFP line. **(D)** The number of reads of ChIP (IP) and input (in) tracks, and log2-transformed ChIP/input ratio tracks (IP/in). Snapshots are shown for the peaks binding at 5’UTR of *pfk13* (PF3D7_1343700), *pfcrt* (PF3D7_0709000), and *pfcrt* (PF3D7_0523000).

As mentioned above, AP2-06B is mutated at high frequency in Southeast Asia where drug resistance originated, which suggested that AP2-06B might be related to the development of drug resistance. AP2-06B is a transcriptional regulatory factor, and understanding the identity and function of its target genes may inform its mechanism of action. Therefore, ChIP-seq assays were performed to explore the role of AP2-06B in transcriptional regulation and whether it might impact expression related to the development of resistance, such as the genes *pfcrt*, *pfk13*, and *pfmdr1*. To achieve this, we first constructed a C-terminal GFP tagged PfAP2-06B transgenic parasite line using the CRISPR/Cas9 gene editing system (**Figure 2A**). Correct editing of the 3D7/PfAP2-06B-GFP strain was verified by collecting genomic DNA from the transgenic parasite line and conventional PCR detection with two sets of primers and sequencing (**Figure 2B**). A perinuclear localization of PfAP2-06B was confirmed by immunofluorescence assay (**Figure 2C**). To investigate transcriptional regulation by PfAP2-06B, ChIP-seq assays were performed on the 3D7/PfAP2-06B-GFP schizonts using antibodies against GFP. The results of ChIP-Seq analysis showed that AP2-06B had a significant binding peak in the 5′ UTR of *pfk13* and *pfcrt*, but not on *pfmdr1* (**Figure 2D**). Therefore, AP2-06B may induce resistance to artemisinin or quinolines by regulating *pfk13* or *pfcrt*; and since K3214R is a mutation located in one of the three predicted DNA-binding AP2 domains of AP2-06B and may be a key mutation in AP2-06B induced resistance.

### 3.3 PfAP2-06B^K3124R^ did not change the expression of *pfk13* or *pfcrt* nor alter susceptibility to artemisinin or quinoline antimalarials

**Figure 3:**
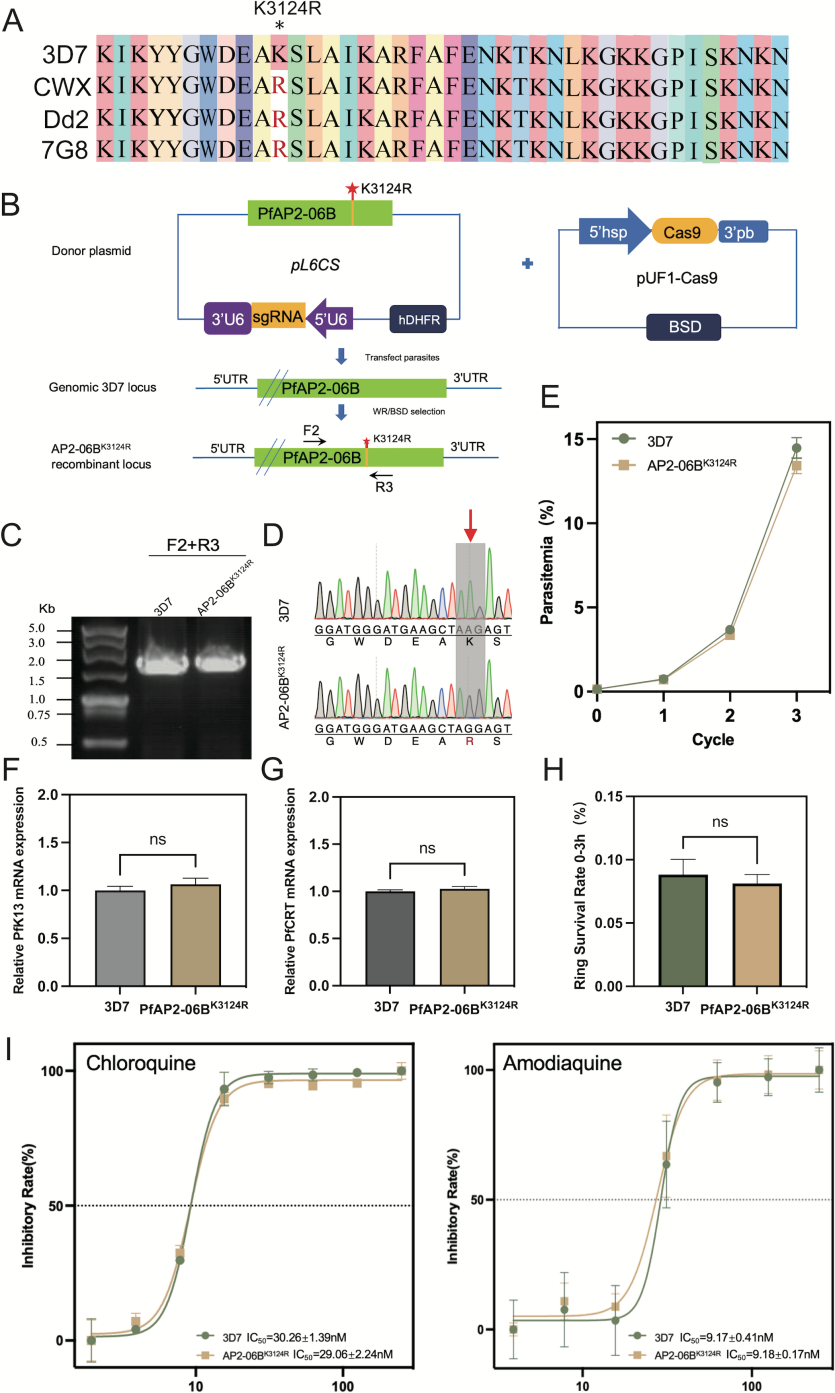
The effect of the K3124R mutation of PfAP2-06B on the expression of *pfk13* and *pfcrt* and the sensitivity of *Plasmodium* to DHA and quinolines. **(A)** Sequence alignment of artemisinin-resistant strain CWX and chloroquine-resistant strains (Dd2 and 7G8) with reference strain Pf3D7. Stars indicate identity with the amino acid in the first sequence. The K3124R mutation was present within the PfAP2-06B AP2 domain of CWX, PfDd2, and Pf7G8. **(B)** Generation of a *pfap2-06b* transgenic parasite line with a K3124R mutation using the CRISPR/Cas9 gene editing system. **(C)** The K3124R mutation of the *pfap2-06b* transgenic line was verified by diagnostic PCR using F1 and R1 primers and **(D)** sequenced. **(E)** Growth curves of 3D7 and the 3D7/AP2-06B^K3124R^ line. **(F)** The mRNA expression of *pfK13* in 3D7 and the 3D7/AP2-06B^K3124R^ line detected by qPCR. **(G)** The mRNA expression of *pfcrt* in 3D7 and the 3D7/AP2-06B^K3124R^ line detected by qPCR. **(H)** The susceptibility of 3D7 and the 3D7/AP2-06B^K3124R^ line to DHA. **(I)** The susceptibility of the 3D7 and 3D7/AP2-06B^K3124R^ lines to the quinoline antimalarials chloroquine (left panel) and amodiaquine (right panel). Comparisons between 3D7 and 3D7/AP2-06B^K3124R^ were performed with the Student’s t-test. *ns* indicates no significance.

The mutation frequency of AP2-06B in Southeast Asia, a region with high drug resistance, is significantly higher than found in other areas. In addition, AP2-06B can bind to the loci of *pfk13* and *pfcrt*, genes which are involved in resistance to artemisinin and quinolines, respectively. As the mutation located in the AP2-06B AP2 domain with a significantly higher mutation frequency in Southeast Asia, we investigated whether K3124R mutation induces resistance to artemisinin and quinolines by regulating *pfk13* and *pfcrt* expression. DNA sequence of the artemisinin-resistant CWX we previously sequenced, and sequences of the quinoline-resistant strains PfDd2 and Pf7G8 were downloaded from PlasmoDB, were analyzed for the genetic diversity of *pfap2-06b*, compared with the Pf3D7 strain as a reference sequence. In contrast to Pf3D7, the K3124R mutation was found within the AP2-06B AP2 domain of the resistant strains CWX, PfDd2, and Pf7G8 (**Figure 3A**).

To determine whether this mutation can alter the expression of *pfk13* and *pfcrt*, as well as cause artemisinin and quinoline resistance in *Plasmodium*, the K3124R mutation was introduced into the endogenous *pfap2-06b* locus of the Pf3D7 strain to generate the edited clone PfAP2-06B ^K3124R^ using CRISPR/Cas9-mediated genome editing (**Figure 3B**). The AP2-06B region containing the mutation of Pf3D7 and PfAP2-06B^K3124R^ was verified by PCR (**Figure 3C**) and sequenced to confirm the successful mutation (**Figure 3D**). To investigate the effect of the K3124R mutation on parasite viability, growth curve assays of PfAP2-06B^K3124R^ were performed, in comparison with the parental 3D7 strain as a wild type control. The result showed that the K3124R mutation had no effect on parasite viability (**Figure 3E**). The sensitivity to artemisinin and quinolines of the Pf3D7 ^K3124R^ and expression of *pfk13* and *pfcrt* were also assayed. The results showed that the K3124R mutation did not change the expression of *pfk13* (**Figure 3F**) or *pfcrt* (**Figure 3G**) and Pf3D7^K3124R^ did not alter susceptibility to DHA (**Figure 3H**) or the quinoline antimalarials chloroquine and amodiaquine (**Figure 3I**).

### 3.4 PfAP2-06B conditional knockout did not change the expression of *pfk13* or *pfcrt* nor alter susceptibility to artemisinin or quinoline antimalarials

**Figure 4:**
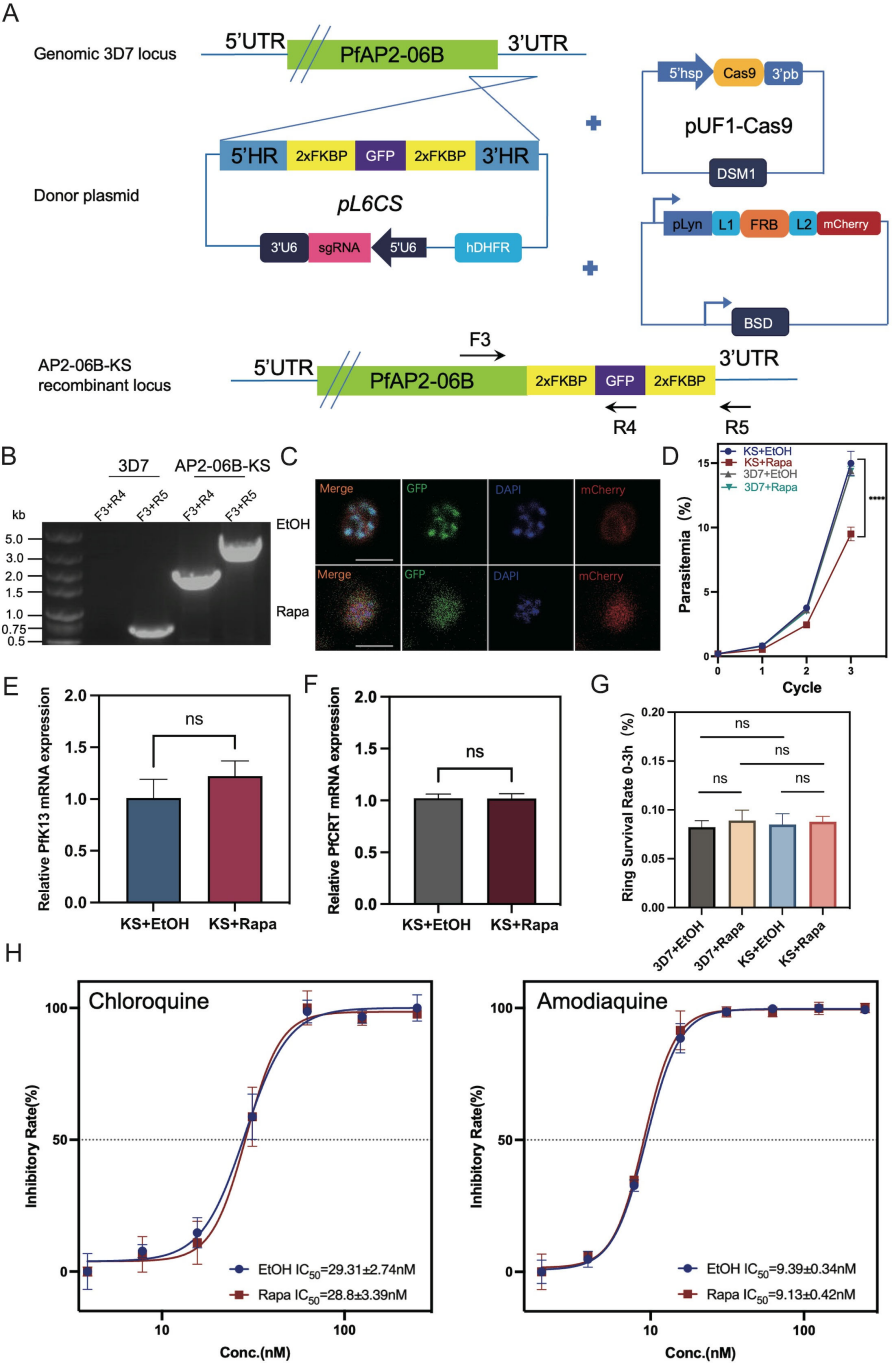
The effect of PfAP2-06B conditional knockdown on the expression of *pfk13* and *pfcrt* and the sensitivity of the transgenic line to DHA and quinolines. **(A)** Incorporation of a PfAP2-06B -2xFKBP-GFP-2xFKBP construct into the PfAP2-06B locus of the wildtype 3D7 line by homologous recombination using the CRISPR/Cas9 gene editing system. **(B)** The KS with lyn mislocalizer inducible knockdown transgenic parasite line was confirmed by diagnostic PCR using primers F1, R1, and R2. **(C)** Representative fluorescence microscopy images of PfAP2-06B-KS late stage schizonts in the absence (EtOH) (upper panel) and presence (Rapa, induced PfAP2-06B KS with lyn mislocalizer) (lower panel) of rapalog. Immunofluorescence assays using anti-GFP revealed a perinuclear distribution of PfAP2-06B in the 3D7/PfAP2-06B-KS line, using anti-mCherry revealed plasma-membrane location of pLyn-FRB-mCherry. **(D)** Growth curves of 3D7 and the 3D7/PfAP2-06B-KS line in the absence (EtOH) or presence of rapalog (Rapa). **(E)** The mRNA expression of *pfK13* in the 3D7/PfAP2-06B-KS line detected by qPCR in the absence (EtOH) or presence (Rapa) of rapalog **(F)**. The mRNA expression of *pfcrt* in the 3D7/PfAP2-06B-KS line detected by qPCR in the absence (EtOH) or presence (Rapa) of rapalog. **(G)** The susceptibility of the 3D7/PfAP2-06B-KS line to DHA in the absence (EtOH) or presence (Rapa) of rapalog. **(H)** The susceptibility of the 3D7/PfAP2-06B-KS line to quinoline antimalarials chloroquine (left panel) and amodiaquine (right panel) in the absence (EtOH) or presence (Rapa) of rapalog. Comparisons between 3D7 and 3D7/PfAP2-06B-KS were performed with the Student’s t-test. ****, P<0.0001, ns indicates no significance.

PfAP2-06B^K3124R^ did not change the expression of *pfk13* or *pfcrt* nor alters susceptibility to DHA or quinoline antimalarials. However, the ChIP-Seq result indicated that AP2-06B had a significant binding peak in the loci of *pfk13* and *pfcrt*, as a transcriptional regulator factor, suggesting that AP2-06B might function as a transcription factor regulating the expression of *pfk13* and *pfcrt*. Therefore, a conditional knockdown strategy was adopted by knock sideways (KS) (**Figure 4A**). KS is based on the ligand-induced dimerization of the proteins FRB and FKBP. The native target is fused with FKBP, whereas FRB is separately expressed with the trafficking information for a different cellular compartment (the ‘mislocalizer’). After addition of the ligand (rapalog) to dimerize FRB and FKBP, the target protein is removed from its site of action by the mislocalizer. This method acts rapidly and avoids the problems of targeting essential genes at the genetic level (Birnbaum et al., 2017). Accordingly, we successfully generated a 3D7/PfAP2-06B-KS transgenic knockdown parasite line and isolated clones by limited dilution cloning. Genomic DNA of the transgenic parasite line was collected, and two sets of primers were used for conventional PCR detection to verify the correct editing of the *pfap2-06b* locus (**Figure 4B**). Immunofluorescence detection confirmed that the localization of PfAP2-06B is transferred from the nucleus to the cell membrane after the addition of rapalog, indicating that AP2-06B knockdown was successful (**Figure 4C**). To investigate the effect of PfAP2-06B on parasite viability, the PfAP2-06B-KS parasite was maintained with rapalog or ethanol (vehicle) to analyze growth, using the parent strain 3D7 as a control. The growth curve analysis showed that the parasitemia of PfAP2-06B-KS strain decreased by 48% treatment with rapalog for a duration of three life cycles compared with the vehicle group, but had no significant effect on the parent strain 3D7 (**Figure 4D**), suggesting that PfAP2-06B is necessary for the growth of *P. falciparum*. The sensitivity of the PfAP2-06B-KS parasite to DHA and quinolines was then assayed in the presence of rapalog versus ethanol; and no effect was observed on susceptibility to DHA (**Figure 4G**) or the quinoline antimalarials chloroquine and amodiaquine (**Figure 4H**). Finally, the expression of *pfk13* and *pfcrt* were quantified, and it was shown that the conditional knockdown of PfAP2-06B did not change the expression of *pfk13* (**Figure 4E**) or *pfcrt* (**Figure 4F**).

## 4 Discussion

It has been reported that mutations in *P. falciparum* AP2 are associated with resistance to antimalarial drugs (Wendler et al., 2014; Cowell et al., 2018). In this study, 6 non-synonymous mutations (R1034C, L2716I, K3124R, N3511D, C3644Y, and M3847V) had allele frequencies greater than 0.05. The mutation frequency of L2716I, K3124R, and M3847V in Southeast Asia, where artemisinin and quinoline resistance originated, was significantly higher than that observed in other regions. The K3124R mutation in PfAP2-06B is located within an AP2 DNA-binding domain, and has a higher mutation frequency in Southeast Asia than in other regions. This indicates that the K3124R mutation may be associated with *Plasmodium* resistance to artemisinin and chloroquine or other quinolines. Further support is provided by the observation that the K3124R mutation is found in the AP2-06B AP2 domain of artemisinin-resistant CWX and chloroquine-resistant strains PfDd2 and Pf7G8.

Because AP2-06B is a transcriptional regulator, its target genes are crucial for characterizing its regulatory mechanism. It was found that AP2-06B can bind to the loci of *pfk13* and *pfcrt*, genes which are related to the resistance of artemisinin and quinolines. However, the creation of a parasite line possessing the AP2-06B K3124R mutation in the sensitive strain 3D7 did not change its response to artemisinin or quinolines nor did it alter the expression of *pfk13* or *pfcrt*. The introduction of the mutation K3124R into AP2-06B did not affect the expression of pfk13 or pfcrt, nor did it alter its sensitivity to artemisinin or quinolines. However, AP-R may still affect the resistance of *Plasmodium* to artemisinin and quinolines by altering the expression of *pfk13* and *pfcrt*; for example, K3124R is the high-frequency mutation located in a PfAP2-06B AP2 domain, and it is possible that there are low-frequency key mutations that are not screened. We cannot determine which mutation plays a key role in *Plasmodium* resistance. Conditional knockdown of AP2-06B was performed to further address the regulatory role of AP2-06B on *pfk13* and *pfcrt* and the resulting resistance of *Plasmodium* to artemisinin and quinolines, and the expression of *pfk13* and *pfcrt* and the resistance of artemisinin and quinolines in the conditional knockdown strain were detected. However, again the results confirmed that the expression of *pfk13* or *pfcrt* was not affected after the conditional knockdown of AP2-06B, and the sensitivity of *Plasmodium* to artemisinin or quinolines was not changed.

Results provided herein suggest that the transcriptional regulator AP2-06B, which has a high frequency mutation in Southeast Asia, does not affect *Plasmodium* susceptibility to artemisinin or quinolines by regulating *pfk13* or *pfcrt* expression, as anticipated. In the research by Cowell AN et al. (Cowell et al., 2018), AP2-06B was mutated in response to three different antimalarial compounds. In addition, another genome-wide association study showed that AP2-06B was also associated with resistance to quinine (Wendler et al., 2014). However, the reported cases are based on long-term drug stress screening and genome-wide analysis; and it still needs to be determined whether these changes are associated with resistance or are related to long-term *in vitro* culture.

In our study, gene editing was used to confirm whether the mutated allele could provide resistance. However, we did not find that the K3124R mutation caused artemisinin or quinoline resistance through gene editing, nor did we observe this result following conditional knockdown of the gene. Genes with a high frequency mutation do not affect resistance, thus there may be other genes that specifically or non-specifically compensate for the fitness caused by the mutations. Alternatively, intergenic and intronic mutations were also common. Although in most cases, resistance was explained by the presence of nonsynonymous coding mutations in the target or resistance genes, intergenic mutations were also common. Likewise, intron variants were more likely to be found in the core genome than in subtelomeric region, suggesting a possible functional role (Cowell et al., 2018). In addition, AP2-06B may not be the only transcription factor that regulates *pfk13* and *pfcrt*, and so it is possible that other transcriptional regulators can also be regulated compensatively after AP2-06B mutation or conditional knockdown.

Given the multigenic nature of resistance, it might be difficult to recreate a resistance phenotype (Plouffe et al., 2008). For example, during the development of resistance to BRD1095, the appearance of non-synonymous changes in phenylalanine tRNA ligases was accompanied by copy number changes at unrelated sites, which may explain why attempts at validation through genome editing were unsuccessful (Cowell et al., 2018). Similarly, in this study, in a *Plasmodium* sensitive strain we mutated and conditionally knocked out AP2-06B, which was predicted to be associated with artemisinin and quinoline resistance, but no change in resistance was observed after mutation or conditional knockdown. It might may be that intergenic and intron mutations and low-frequency non-coding mutations are involved in regulating the development of resistance; or other unknown changes that that can specifically or non-specifically compensate for the loss of fitness caused by the AP2-06B mutation or conditional knockdown conferring resistance. In conclusion, whether AP2-06B regulates the development of artemisinin or quinoline resistance remains to be studied.

## Data Availability

The data presented in the study are deposited in the SRA repository, accession number PRJNA1195675.
