# Supplementary figures and images for: Plasmodium falciparum transcription factor AP2-06B is mutated at high frequency in Southeast Asia but does not associate with drug resistance
Source: Front Cell Infect Microbiol. 2025 Jan 6;14:1521152. doi: 10.3389/fcimb.2024.1521152 (PMC11744005; doi:10.3389/fcimb.2024.1521152)

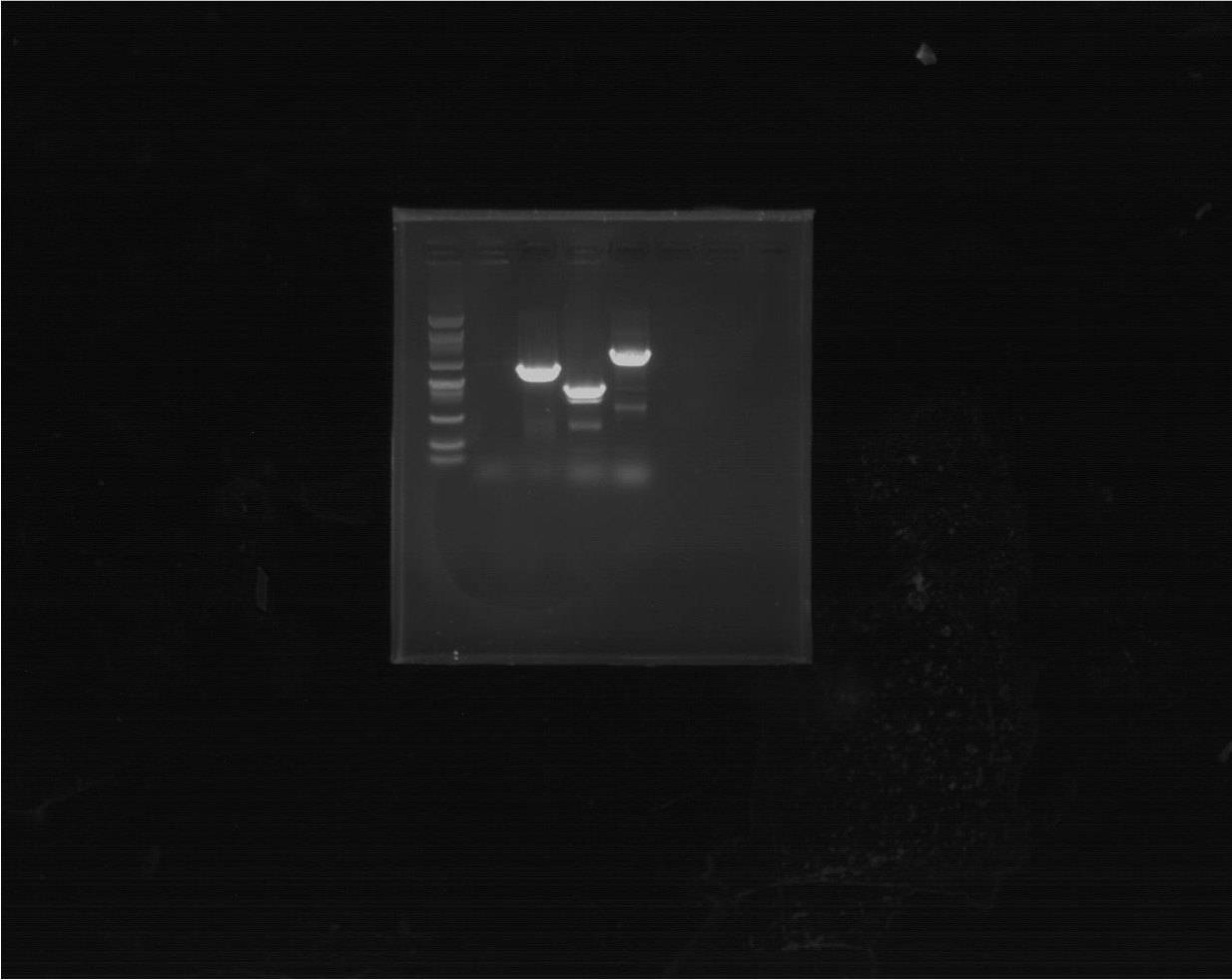

Supplement: Supplementary file 1 [file Image1.jpeg]

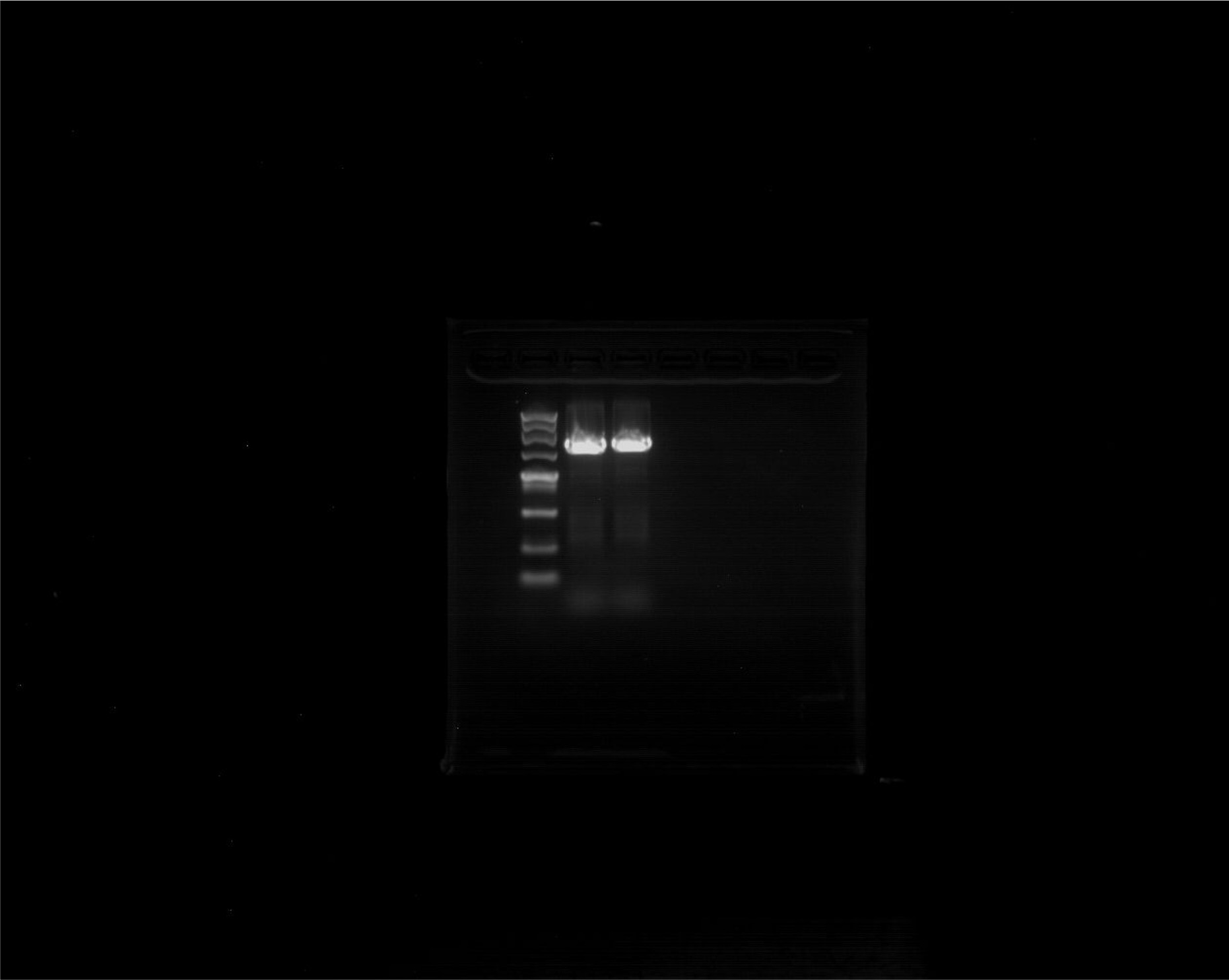

Supplement: Supplementary file 2 [file Image2.jpeg]

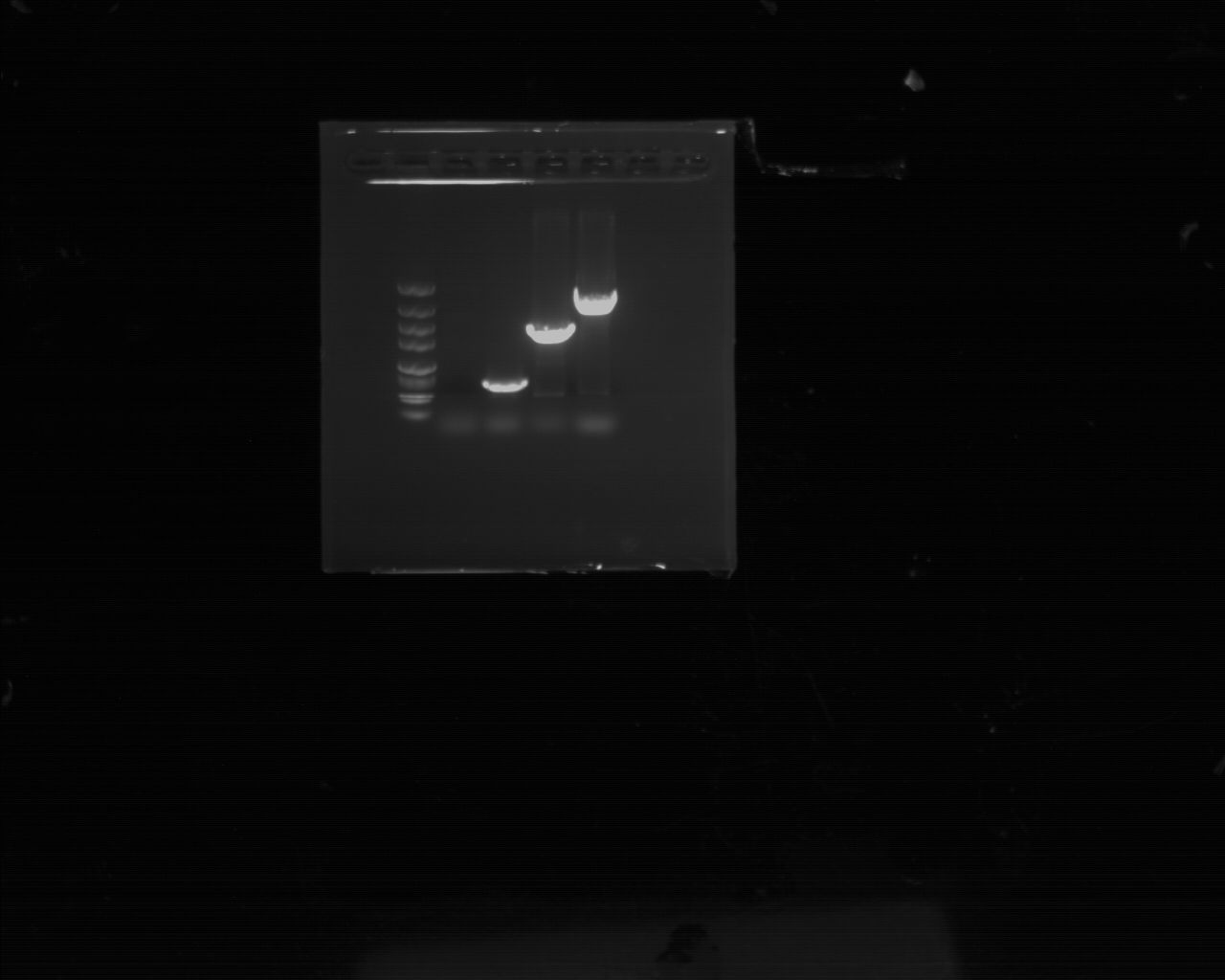

Supplement: Supplementary file 3 [file Image3.jpeg]
