# Supplementary material for: Plasmodium falciparum transcription factor AP2-06B is mutated at high frequency in Southeast Asia but does not associate with drug resistance
Source: Front Cell Infect Microbiol. 2025 Jan 6;14:1521152. doi: 10.3389/fcimb.2024.1521152 (PMC11744005; doi:10.3389/fcimb.2024.1521152)
Supplement: Supplementary file 4 [file Table1.docx]

| Table S1 \| Primers used for construction | |  |
| --- | --- | --- |
| PrimerID | Sequence | Strand |
| AP2-R-ty1-GFP-LHRF | CCTAGTCTAGGGCGCGCCCATATACTTAATATAACCAAAGAAATAACAG | Forward |
| AP2-R-ty1-GFP-sgRNAR | CCTCGCCCTCATTTCGATAGCTAATTTTTTACTTAAG | Reverse |
| AP2-R-ty1-GFP-sgRNAF | ATCGAAATGAGGGCGAGGATGACTGGAGAATGTTTAATTTCAG | Forward |
| AP2-R-ty1-GFP-LHRR | GTCAACCGCTGCGGCAGCATTACTCATAGTTCCTTTTTTTGTTG | Reverse |
| AP2-R-ty1-GFP-F | GCTGCCGCAGCGGTTGACGCAGCAGCAGCTCTCGAGGAAGTACATACTAACCAAGATCC | Forward |
| AP2-R-ty1-GFP-R | GCGGCCGCTTATTTGTATAGT | Reverse |
| AP2-R-ty1-GFP-RHRF | ATACAAATAAGCGGCCGCTAATAATAAAATATATATACAAAGGTGAAATTATATCAC | Forward |
| AP2-R-ty1-GFP-RHRR | TTTTACAAAATGCTTAAGTTGCTGGTGCAGTTAATATTG | Reverse |
| AP2-R-ty1-sgRNAF | TATTAAGTATATAATATTGAAATGAGAGCAAGAATGAC | Forward |
| AP2-R-ty1-sgRNAR | TATTTCTAGCTCTAAAACGTCATTCTTGCTCTCATTTC | Reverse |
| AP2-R-K3124R-1F | GCGGCCCTAGTCTAGGGCGCGCCGACAAATACGTTAACAAATATGCA | Forward |
| AP2-R-K3124R-2R | CCTGGCTTTGATAGCTAGACTCCTAGCTTCATCCCATCCAT | Reverse |
| AP2-R-K3124R-2F | GGAGTCTAGCTATCAAAGCCAGGTTTGCTTTTGAAAATAAGAC | Forward |
| AP2-R-K3124R-1R | AATTTTTTTTACAAAATGCTTAAGGGTTGTGTATCATCAATATGGT | Reverse |
| AP2-R-K3124R-sgRNAF | TAAGTATATAATATTAGAGTCTCGCTATAAAAGCTGTTTTAGAGCTAGAA | Forward |
| AP2-R-K3124R-sgRNAR | TTCTAGCTCTAAAACAGCTTTTATAGCGAGACTCTAATATTATATACTTA | Reverse |
| AP2-R-sideway-LHRF | CCTAGTCTAGGGCGCGCCCATATACTTAATATAACCAAAGAAATAACAG | Forward |
| AP2-R-sideway-mR | CCTCGCCCTCATTTCGATAGCTAATTTTTTACTTAAG | Reverse |
| AP2-R-sideway-mF | ATCGAAATGAGGGCGAGGATGACTGGAGAATGTTTAATTTCAG | Forward |
| AP2-R-sideway-LHRR | GTCAACCGCTGCGGCAGCATTACTCATAGTTCCTTTTTTTGTTG | Reverse |
| AP2-R-sideway-Ty1F | GCTGCCGCAGCGGTTGACGCAGCAGCAGCTCTCGAG | Forward |
| AP2-R-sideway-FKBP-F | GACGCAGCAGCAGCTCTCGAGTCAGGATTGAGATCAAGATCTG | Forward |
| AP2-R-sideway-FKBP-R | TATATTTTATTATTAGCGGCCGCCTATTCCAGTTTCAAAAGTTCGAC | Reverse |
| AP2-R-sideway-RHRF | GCGGCCGCTAATAATAAAAT | Forward |
| AP2-R-sideway-RHRR | TTTTACAAAATGCTTAAGTTGCTGGTGCAGTTAATATTG | Reverse |
| AP2-R-ty1-sgRNAF | TATTAAGTATATAATATTGAAATGAGAGCAAGAATGAC | Forward |
| AP2-R-ty1-sgRNAR | TATTTCTAGCTCTAAAACGTCATTCTTGCTCTCATTTC | Reverse |
