# Supplementary material for: Plasmodium falciparum transcription factor AP2-06B is mutated at high frequency in Southeast Asia but does not associate with drug resistance
Source: Front Cell Infect Microbiol. 2025 Jan 6;14:1521152. doi: 10.3389/fcimb.2024.1521152 (PMC11744005; doi:10.3389/fcimb.2024.1521152)
Supplement: Supplementary file 5 [file Table2.docx]

| Table S3 \| Formulation of SYBR Green I lysis buffer solution (26 mL) | |
| --- | --- |
| Component | Volume（mL） |
| 1M Tris-HCL pH 7.5 | 0.52 |
| 0.5M EDTA pH 8.0 | 0.26 |
| 3% saponin | 0.06934 |
| Triton X-100 | 2.08 |
| SYBR Green I | 0.0052 |
| 1×PBS pH 7.4 | 23.064 |
