# Supplementary material for: Plasmodium falciparum transcription factor AP2-06B is mutated at high frequency in Southeast Asia but does not associate with drug resistance
Source: Front Cell Infect Microbiol. 2025 Jan 6;14:1521152. doi: 10.3389/fcimb.2024.1521152 (PMC11744005; doi:10.3389/fcimb.2024.1521152)
Supplement: Supplementary file 6 [file Table3.docx]

| Table S4 \| Real-time Quantitative PCR assays primers | | |
| --- | --- | --- |
| PrimerID | Sequence | Strand |
| SSP2-F | TAACTTGTATGCTGATTCTGC | Forward |
| SSP2-R | GATTTCTCTTTTTCTTGACC | Reverse |
| FBA-F | TGTACCACCAGCCTTACCAG | Forward |
| FBA-R | TTCCTTGCCATGTGTTCAAT | Reverse |
| Seryl-tRNA-F | AAGTAGCAGGTCATCGTGGTT | Forward |
| Seryl-tRNA-R | TTCGGCACATTCTTCCATAA | Reverse |
| CRT-F | GTTCTTGTCTTGGTAAATGTGCTCA | Forward |
| CRT-R | CAATTTTGTTTAAAGTTCTTTTAGCAA | Reverse |
| K13-F | GGAAAGAGTACGATTGTACA | Forward |
| K13-R | CTTCATCAAATCGTTTCCTATG | Reverse |
