# Supplementary material for: Plasmodium falciparum transcription factor AP2-06B is mutated at high frequency in Southeast Asia but does not associate with drug resistance
Source: Front Cell Infect Microbiol. 2025 Jan 6;14:1521152. doi: 10.3389/fcimb.2024.1521152 (PMC11744005; doi:10.3389/fcimb.2024.1521152)
Supplement: Supplementary file 7 [file Table4.docx]

| Table 2 \| Primers used for verification | | |
| --- | --- | --- |
| PrimerID | Sequence | Strand |
| F1 | GATCCTGTTAAAGTACAGAGAG | Forward |
| R1 | cattgtgtgagttatagttgtattcc | Reverse |
| R2 | ttacctgaacagttcagg | Reverse |
| F2 | TGGTTCATTGTGTGATGT | Forward |
| R3 | GATCAGATTGGTCATTTTTATG | Reverse |
| F3 | GATCCTGTTAAAGTACAGAGAG | Forward |
| R4 | GTGTCTTGTAGTTCCCGT | Reverse |
| R5 | ttacctgaacagttcagg | Reverse |
